# Antibody against Microbial Neuraminidases Recognizes Human Sialidase 3 (NEU3): the Neuraminidase/Sialidase Superfamily Revisited

**DOI:** 10.1128/mBio.00078-17

**Published:** 2017-06-27

**Authors:** Chiguang Feng, Jihong Li, Greg Snyder, Wei Huang, Simeon E. Goldblum, Wilbur H. Chen, Lai-Xi Wang, Bruce A. McClane, Alan S. Cross

**Affiliations:** a Center for Vaccine Development, University of Maryland School of Medicine, Baltimore, Maryland, USA; b Department of Microbiology and Molecular Genetics, University of Pittsburgh School of Medicine, Pittsburgh, Pennsylvania, USA; c Institute of Human Virology and Department of Microbiology and Immunology, University of Maryland School of Medicine, Baltimore, Maryland, USA; d Department of Medicine, University of Maryland School of Medicine, Baltimore, Maryland, USA; University of Maryland, College Park

**Keywords:** Neu3, inflammation, innate immunity, neuraminidase, sialidase

## Abstract

Neuraminidases (NAs) are critical virulence factors for several microbial pathogens. With a highly conserved catalytic domain, a microbial NA “superfamily” has been proposed. We previously reported that murine polymorphonuclear leukocyte (PMN) sialidase activity was important in leukocyte trafficking to inflamed sites and that antibodies to *Clostridium perfringens* NA recognized a cell surface molecule(s), presumed to be a sialidase of eukaryotic origin on interleukin-8-stimulated human and murine PMNs. These antibodies also inhibited cell sialidase activity both *in vitro* and, in the latter instance, *in vivo*. We therefore hypothesized that mammalian sialidases share structural homology and epitopes with microbial NAs. We now report that antibodies to one of the isoforms of *C. perfringens* NA, as well as anti-influenza virus NA serum, recognize human NEU3 but not NEU1 and that antibodies to *C. perfringens* NA inhibit NEU3 enzymatic activity. We conclude that the previously described microbial NA superfamily extends to human sialidases. Strategies designed to therapeutically inhibit microbial NA may need to consider potential compromising effects on human sialidases, particularly those expressed in cells of the immune system.

## IMPORTANCE

We previously reported that sialidase activity of human neutrophils plays a critical role in the host inflammatory response. Since the catalytic domains of microbial neuraminidases are highly conserved, we hypothesized that antibodies against *Clostridium perfringens* neuraminidase might inhibit mammalian sialidase activity. Before the recognition of four mammalian sialidase (*Neu*) isoforms, we demonstrated that anti-*C. perfringens* neuraminidase antibodies inhibited human and murine sialidase activity *in vivo* and *in vitro*. We now show that the antibodies to microbial neuraminidase (*C. perfringens* and influenza virus) recognize human NEU3, which is important for neural development and cell signaling. Since many microbes that infect mucosal surfaces express neuraminidase, it is possible that the use of sialidase inhibitors (e.g., zanamivir), might also compromise human sialidase activity critical to the human immune response. Alternatively, sialidase inhibitors may prove useful in the treatment of hyperinflammatory conditions.

## INTRODUCTION

Microbial neuraminidases (NAs), enzymes that cleave sialic acid from cell surface glycoconjugates, are important virulence factors for pathogens, particularly those that target mucosal surfaces. For example, influenza virus NA is critical to its infective cycle and is therefore a target of antiviral therapy (1). *Pseudomonas aeruginosa* and *Streptococcus pneumoniae* rely on NAs to colonize the mammalian host (2). While microbial NA amino acid sequences are <40% identical, their catalytic domain is highly conserved and they share a “six-bladed propeller fold” architecture and conserved motifs called “Asp boxes” and “FRIP regions” (3). On the basis of these observations, a microbial NA “superfamily” has been proposed (3).

We previously reported that the sialidase activity in human polymorphonuclear leukocytes (PMNs) played a critical role in the host response to infection and inflammation (4, 5) and that its activity was upregulated following PMN activation both *in vitro* and *in vivo* (5–7). We also observed that murine PMN sialidase activity was important in leukocyte trafficking to inflamed sites and hypothesized that since the catalytic domain of microbial NAs was highly conserved, antibodies against microbial NAs might recognize and subsequently inhibit mammalian sialidase activity. Indeed, we demonstrated that antibodies to *Clostridium perfringens* NA recognized a cell surface molecule(s) on both human and murine PMNs after interleukin-8 stimulation *in vitro* and these same antibodies inhibited PMN sialidase activity both *in vitro* and *in vivo* (4, 5). The targeted molecule(s), presumed to be human sialidase, was not identified.

Since that report, four sialidases with distinct cellular localizations and likely different substrate preferences and cellular functions have been identified in humans and mice (8–11). The most abundant, lysosomal sialidase (NEU1), associates with other proteins to form a multienzyme complex (9, 12). Membrane-associated sialidase (NEU3) is a protein that preferentially desialylates gangliosides (13, 14) and perhaps selected surface glycoproteins (15). NEU3 promotes cell adhesion to laminins and integrin-mediated cell proliferation (16). Cytosolic sialidase (NEU2) can desialylate both glycoproteins and gangliosides and may have a role in myoblast differentiation (17). NEU4, which is located in the lysosomal and mitochondrial lumena, may be important for ganglioside catabolism and lysosomal storage at these sites and in neuronal differentiation (18), but its functional effect on glycoproteins is unknown.

Here, we report that the anti-*C. perfringens* NA antibody previously examined (4) and antisera to specific influenza virus NAs all recognize human NEU3 but not NEU1. Since human immune cells and respiratory epithelia (19) have sialidase activity, these data may have significant implications for the desirability of inhibiting microbial sialidase activity without considering its impact on host sialidases, which are important components of the host immune response.

## RESULTS

### Anti-*C. perfringens* NA rabbit serum recognizes rNEU3 but not rNEU1.

**FIG 1:**
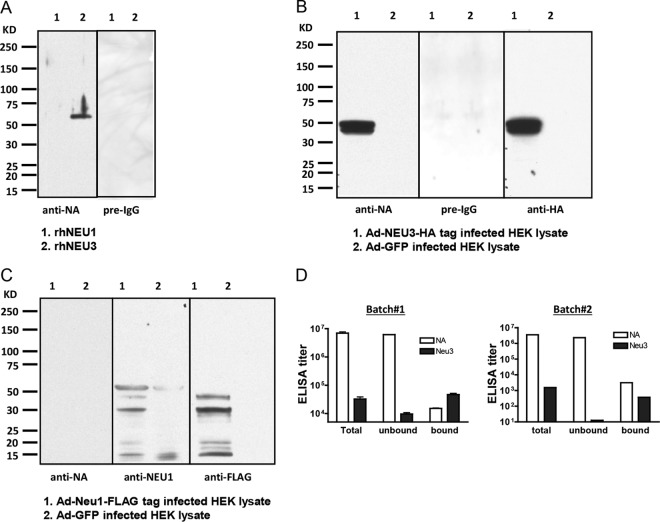
Anti-*C. perfringens* NA rabbit antibody recognizes human NEU3 but not NEU1. (A) rhNEU1 (lane 1) and rhNEU3 (lane 2) proteins were resolved by SDS-PAGE and immunoblotted with rabbit anti-*C. perfringens* NA antibody (anti-NA) or preimmune antibody (pre-IgG). (B) HEK293T cells were infected with Ad-NEU3-HA (lane 1) or control Ad-GFP (lane 2) (multiplicity of infection, 20) and lysed, and the lysates (100 µg) were processed for immunoblotting with rabbit anti-NA antibody (anti-NA; 1:500), preimmune antibody (pre-IgG), or anti-HA tag antibody (anti-HA; 1:500). (C) HEK293T cells were infected with recombinant Ad-NEU1-FLAG (lanes 1) or control Ad-GFP (lanes 2) (multiplicity of infection, 20) and lysed, and the lysates were processed for immunoblotting with rabbit anti-*C. perfringens* NA antibody (anti-NA), anti-NEU1 antibody (1:500) (anti-NEU1), or anti-FLAG tag antibody (1:500) (anti-FLAG). Representative data from three independent experiments are shown. (D) Rabbit anti-*C. perfringens* NA serum was passed through the rhNEU3 affinity column and separated into bound and unbound fractions. The IgG antibody titers against either *C. perfringens* NA (NA) or rhNEU3 (Neu3) in the unfractionated antibody pool (Total), bound eluate fraction (bound), and unbound fraction (unbound) were quantified by ELISA. Two independent experiments with two different preparations of rabbit antisera are presented.

We previously demonstrated that anti-*C. perfringens* NA antibody recognized a cell surface protein(s) on PMNs and inhibited PMN migration (4, 5). We hypothesized that this antibody to bacterial NA would recognize one or more mammalian sialidases. Of the four sialidases identified in humans, NEU1 and NEU3 have been shown to modulate sialic acids on the cell surface (9, 12, 14, 15). To study the function of sialidase, we synthesized human recombinant NEU1 (rNEU1) and rNEU3 and constructed adenovirus (Ad) vectors encoding the human genes for FLAG-tagged NEU1 (Ad-NEU1-FLAG) and hemagglutinin (HA)-tagged NEU3 (Ad-NEU3-HA). To establish whether human NEU1 and/or NEU3 is recognized by anti-*C. perfringens* NA serum, we probed blots of the recombinant human NEU1 (rhNEU1) and rhNEU3 proteins. Interestingly, anti-*C. perfringens* NA serum recognized the rhNEU3 but not the rhNEU1 protein (Fig. 1A); preimmune serum (as a negative control) did not recognize either. We next overexpressed the NEU3 or NEU1 protein in HEK293T cells infected with either Ad-NEU3-HA (Fig. 1B) or Ad-NEU1-FLAG (Fig. 1C). Empty-vector virus (Ad-green fluorescent protein [GFP])-infected cells were included as a negative control in both experiments. Anti-*C. perfringens* NA antibody recognized a double band at 50 kDa from Ad-NEU3-HA-infected cells that was also recognized by an anti-HA tag antibody but not by preimmune serum (Fig. 1B). These results indicate that the anti-*C. perfringens* NA antibody recognized human NEU3.

In contrast, no band was detected in blots of lysates of Ad-NEU1-FLAG-infected cells probed with the same anti-*C. perfringens* NA antibody (Fig. 1C). Immunoblotting with either an anti-NEU1 or an anti-FLAG tag antibody revealed both NEU1-immunoreactive and FLAG tag-immunoreactive bands (Fig. 1C). These combined data indicate that NEU1 was expressed in Ad-NEU1-FLAG-infected cells but was not recognized by the anti-*C. perfringens* NA antibody.

### A fraction of anti-*C. perfringens* NA serum recognizes NEU3.

Since anti-*C. perfringens* NA serum recognized rhNEU3 under denaturing conditions in a Western blot assay, we next determined if the antiserum might also recognize NEU3 in its natural form in an enzyme-linked immunosorbent assay (ELISA). The anti-*C. perfringens* NA serum had a robust ELISA titer against *C. perfringens* NA, which was used as the antigen (3.6 × 10^6^ to 43 × 10^6^ ELISA units [EU]/ml); this antiserum also contained an antibody against rhNEU3 (1.5 × 10^3^ to 48 × 10^3^ EU/ml) (Table 1). Since there is <40% homology between the *C. perfringens* NA and rhNEU3, the difference between the anti-*C. perfringens* NA and NEU3 antibody titers can be attributed to the likelihood that the anti-*C. perfringens* NA antibody has epitopes on the *C. perfringens* NA that are not present on NEU3. We asked whether there might be multiple targets for the rabbit anti-*C. perfringens* NA polyclonal antibody in the *C. perfringens* NA preparation and how much of the total anti-*C. perfringens* NA antibody was directed against NEU3. To address this question, we passed the antiserum through an rhNEU3 affinity column and assayed the bound (eluate) and unbound (pass through) fractions by ELISA against *C. perfringens* NA (type V) or rhNEU3. The rhNEU3-unbound portion of the initial antiserum accounted for most of the anti-*C. perfringens* NA titer but very little of the anti-NEU3 titer, while the rhNEU3-bound portion of the antiserum (eluate) accounted for only a small portion of the anti-*C. perfringens* NA titer but contained most of the anti-NEU3 antibody titer (Fig. 1D). These data suggest that while NA immunization elicited a robust antibody response to NA, the immunogen, only a small portion of the antibodies cross-reacted with rhNEU3.

**TABLE 1:** Anti-*C. perfringens* NA serum recognizes *C. perfringens* NA and rhNEU3

**Antigen**^a^	**ELISA titer**^b^	
	*C. perfringens* **NA**	**rhNEU3**
Batch 1	4.3 × 10^7^	4.8 × 10^4^
Batch 2	6.9 × 10^6^	3.2 × 10^4^
Batch 3	3.6 × 10^6^	1.5 × 10^3^

^a^ Sera from three immunized rabbits were tested for their titers of antibodies to either *C. perfringens* NA or rhNEU3. The background reactivity with preimmune serum produced an ELISA titer of <25.

^b^ ELISA titers were calculated through linear regression as the inverse of the serum dilution that produced an absorbance value of 0.2 above the blank.

### Anti-*C. perfringens* NA rabbit serum recognizes NanI.

**FIG 2:**
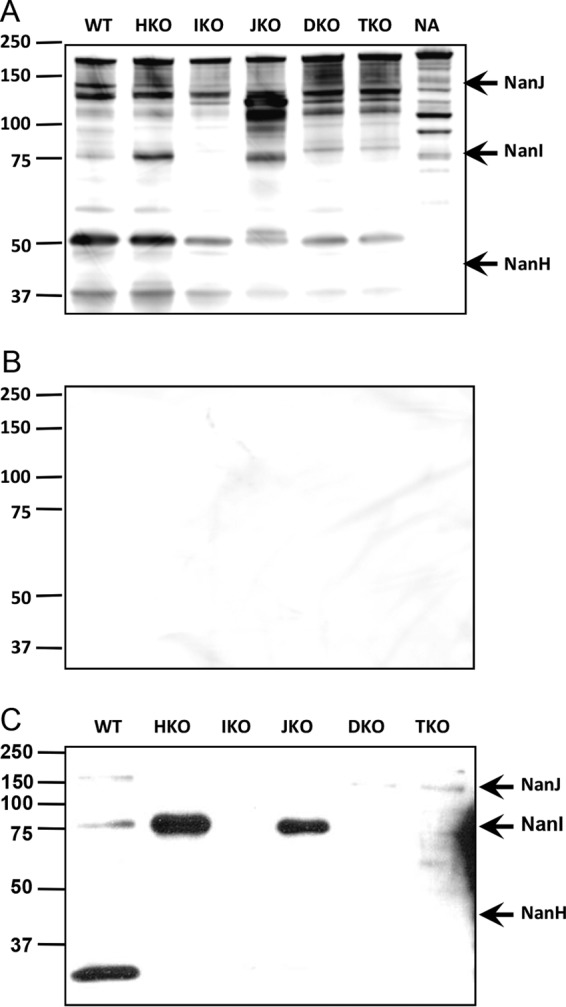
Rabbit anti-*C. perfringens* NA antibody recognizes *C. perfringens* NanI. (A) Cell lysates of a culture of wild-type (WT) *C. perfringens*, single-KO strains each lacking one of three NA isoforms (NanH [HKO], NanI [IKO], or NanJ [JKO]), a double-KO strain lacking NanJ and NanI (DKO), or a triple-KO strain lacking all of the isoforms (TKO) were prepared as described previously (20) and processed for immunoblotting with rabbit anti-*C. perfringens* NA antibody (1:500). *C. perfringens* NA type V (NA), the immunogen, was included as a positive control. (B) Cell lysates were subjected to immunoblotting with preimmune rabbit antibody. (C) Cell lysates of a bacterial culture from wild-type and KO *C. perfringens* strains were subjected to immunoblotting with NEU3 affinity-purified antibody (1:500). Representative data from two independent experiments are shown.

*C. perfringens* has three NA isoforms: NanI at 77 kDa, NanH at 47 kDa, and NanJ at 130 kDa (20). Because we immunized rabbits with *C. perfringens* NA (Sigma), a mixture of the three isoforms, we wanted to identify whether the anti-*C. perfringens* NA antibody recognized one or all of the isoforms. We recently generated single-knockout (KO) mutants lacking NanH (BMC203), NanI (BMC202), or NanJ (BMC201); a double-KO strain lacking NanI and NanJ (BMC204); and a triple-KO strain (BMC205) lacking all three isoforms (20). We prepared the culture supernatant from wild-type or KO bacterial strains as described previously (20), after which we processed the supernatant for immunoblotting with immune rabbit serum or preimmune serum (as a negative control). Multiple bands from the NA antigen preparation were recognized by the anti-*C. perfringens* NA serum, including one at 77 kDa and one at 130 kDa (Fig. 2A, lane NA). No 77-kDa band was detectible in blots of the NanI KO (lane IKO), double-KO (lane DKO), or triple-KO (lane TKO) bacterial strains, indicating that the anti-NA serum recognized primarily the NanI peptide. The specificities for NanH and NanJ were less conclusive. Preimmune serum did not recognize any of the above proteins (Fig. 2B).

To test if the antibodies that cross-reacted with NEU3 also recognized *C. perfringens* NA, we immunoblotted the culture supernatant from wild-type or KO bacterial strains with the eluted antibodies. The 77-kDa band detected in the wild-type strain and two KO strains was absent from the NanI KO (lane IKO), double-KO (lane DKO), and triple-KO (lane TKO) strains (Fig. 2C). There was no reactivity with either NanJ or NanH. These data suggest that the NEU3-cross-reactive antibody interacts with NanI and NEU3 has epitopes in common with NanI.

**FIG 3:**
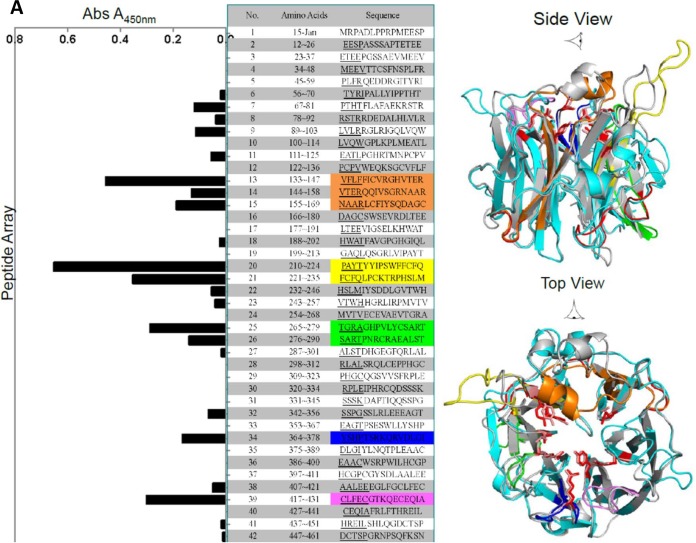

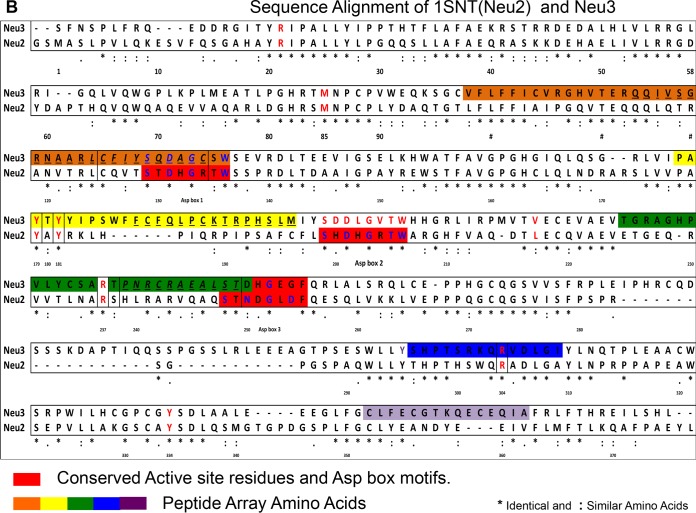
Rabbit anti-*C. perfringens* NA antibody recognizes human NEU3. (A, left) Titers of antibodies against an array of synthesized 15-mer peptides of rhNEU3 were measured by ELISA. The pooled data reflect the average of three independent experiments, and the background absorbance with preimmune antibody was subtracted. (A, right) Cartoon representation of the model of human NEU3 (cyan) based on the crystal structure of human NEU2 (grey) (PDB code 1SO7 [sugar-induced form]). Amino acid 15-mers identified in a peptide array are orange, yellow, green, blue, and violet from the amino terminus to the carboxyl terminus. (B) Clustal W sequence alignment of human NEU2 and NEU3. Highlighted in red are conserved active-site and Asp motif amino acids. About 15-mer peptides identified in peptide array experiments are orange, yellow, green, blue, and violet from the amino terminus to the carboxyl terminus. Asterisks and colons indicate identical and similar residues, respectively.

To map the linear epitopes in NEU3 recognized by anti-*C. perfringens* NA antibodies, we generated a 15-mer peptide array, each peptide with four or five amino acids overlapping at each end, and performed an ELISA. The ELISA revealed five major linear regions (13 to 15 [orange], 20 and 21 [yellow], 25 and 26 [green], 34 [blue], and 39 [purple]) that reacted with the anti-*C. perfringens* NA antibodies (Fig. 3A). To determine if one or more of these five regions would reside in close proximity in a three-dimensional structure, we performed a BLAST search by using the protein sequence for human Neu3 against known structures deposited in the Protein Data Bank (PDB) of structures. The crystal structure of human Neu2 (PDB code 1SNT) was identified as the highest-ranking homologous structure, with 41% identity and an E value of 9e-83. A sequence alignment of human NEU2 and NEU3 with CLUSTAL W identified conserved ligand binding loop, active-site amino acids and Asp box motifs (Fig. 3B). Asterisks are below the amino acids that are identical in Neu2 and Neu3, while colons are below the amino acids that are similar but not identical. An ~30-amino-acid insertion predicted to be high helical for NEU3 was observed between amino acids 291 and 321 (light yellow) in comparison to NEU2. These regions appear in close proximity near the catalytic pocket in a molecular model of NEU3 (Fig. 3A). The colored highlights in the structural model correspond to the colored sequences in the middle panel, while the red sequences correspond to the conserved active-site residues and Asp box motifs also shown in Fig. 3B.

### Anti-*C. perfringens* NA antibodies inhibit sialidase activities of NEU3.

**FIG 4:**
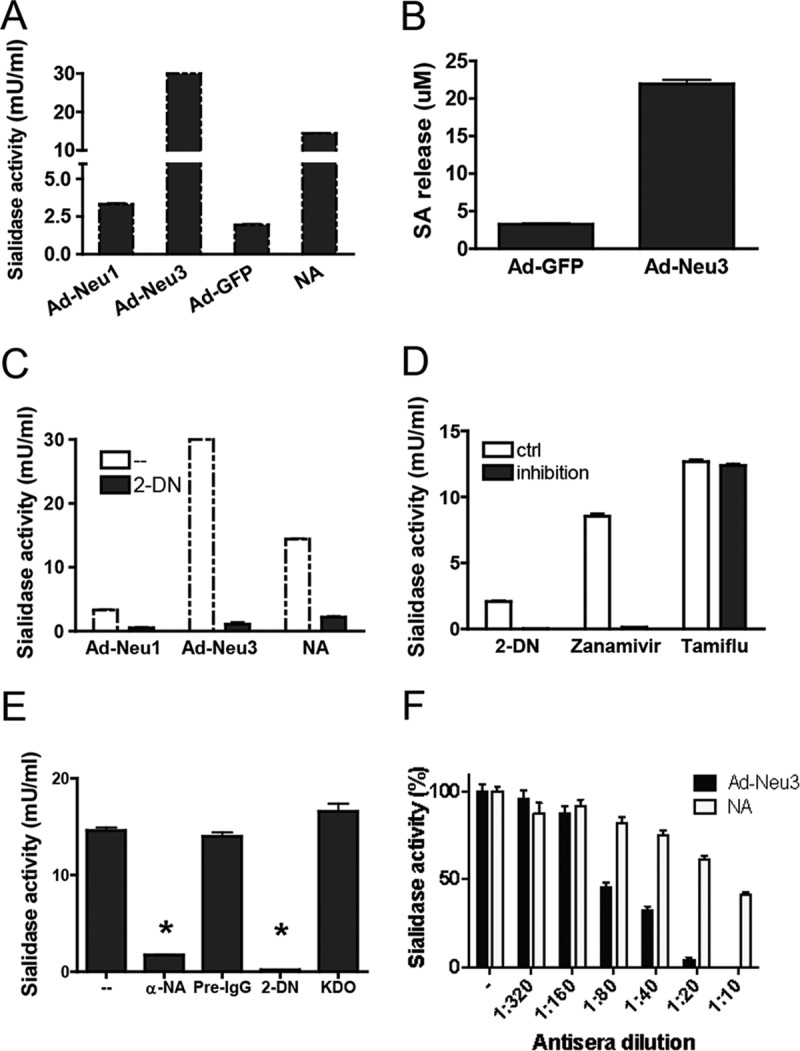
Rabbit anti-*C. perfringens* NA antibody inhibits human NEU3 sialidase activity. (A) HEK293T cells were infected with Ad-NEU1-FLAG (Ad-Neu1), Ad-NEU3-HA (Ad-Neu3), or control virus Ad-GFP (multiplicity of infection, 20). Infected HEK293T cells (2 × 10^6^) were lysed in 200 µl of reaction buffer with Triton X-100, and the sialidase activities in cell lysates were determined with 4-MUNANA as the substrate. A sample with a specific amount of NA was included as a positive control. (B) The sialidase activities in lysates of Ad-NEU3-HA (Ad-Neu3)- or Ad-GFP-infected HEK293T cells (2 × 10^6^) were determined with mixed gangliosides as the substrate. (C) The sialidase activities in lysates of Ad-NEU1-FLAG (Ad-Neu1)- or Ad-NEU3-HA (Ad-Neu3)-infected HEK293T cells in the absence (−) or presence of the NA inhibitor 2-DN were determined with 4-MUNANA as the substrate. A sample with a specific amount of NA was included as a positive control. (D) The sialidase activities in lysates of Ad-NEU3-HA-infected HEK293T cells in the absence (ctrl) or presence (inhibition) of specific NA inhibitors were determined with 4-MUNANA as the substrate. (E) Ad-NEU3-HA-infected HEK293T cells (2 × 10^6^) were lysed in 200 μl of reaction buffer with Triton X-100 (1%) and incubated for 15 min with 5 μl of rabbit anti-*C. perfringens* NA serum (α-NA), preimmune antibody (Pre-IgG), 2-DN (10 mg/ml; final concentration, 250 μg/ml), or KDO (10 mg/ml; final concentration, 250 μg/ml), and the sialidase activity was determined with 4-MUNANA as the substrate. *, *P* < 0.01 versus the untreated control (−). (F) The lysate of Ad-NEU3-HA-infected HEK293T cells (1 × 10^6^) or *C. perfringens* NA (0.2 mU) in 200 µl of reaction buffer with Triton X-100 (0.1%) was incubated for 15 min with serial dilutions of anti-*C. perfringens* NA antisera (Table 1, batch 2), and the sialidase activity was determined with 4-MUNANA as the substrate. The sialidase activity at each antiserum dilution is expressed as a percentage of the sialidase activity measured in the absence of antisera (−). Representative data from three independent experiments are shown.

As we demonstrated that anti-*C. perfringens* NA antibodies recognized rhNEU3 (Fig. 1 and 3), we asked whether the antibacterial NA antibody exerts any inhibitory activity against NEU3 sialidase activity. Purified rhNEU1 and rhNEU3, expressed from an *Escherichia coli* culture, did not show any sialidase activity (data not shown). To generate functional sialidases, HEK293T cells were infected with Ad-NEU1-FLAG, Ad-NEU3-HA, or mock virus Ad-GFP. The sialidase activities in HEK293T cell lysates were assayed with 2′-(4-methylumbelliferyl)-α-d-*N*-acetylneuraminic acid sodium salt hydrate (4-MUNANA) as the substrate. Ad-GFP-infected cells had modest amounts of sialidase activity (Fig. 4A), similar to the level in uninfected cells (data not shown), suggesting some background level of sialidase activity in HEK293T cells. Compared to Ad-GFP-infected cells, both Ad-NEU1-FLAG- and Ad-NEU3-HA-infected cells demonstrated higher sialidase activities (0.7- and 14.4-fold, respectively, Fig. 4A). Using gangliosides, a preferred substrate, Ad-NEU3-HA-infected cells demonstrated a nearly 6-fold increase in sialidase activity (Fig. 4B). Furthermore, the Ad-NEU1 and Ad-NEU3 sialidase activities could be inhibited 6- and 27-fold, respectively, by the sialidase inhibitor 2,3-dehydro-2-deoxy-*N*-acetylneuraminic acid (2-DN) (Fig. 4C), as well as by 4-guanidino-2-deoxy-2,3-dehydro-*N*-acetylneuraminic acid (zanamivir) (Fig. 4D).

The sialidase activity of lysates of Ad-NEU3-HA-infected HEK293T cells was diminished by anti-*C. perfringens* NA antibodies (Fig. 4E) and 2-DN but not by preimmune serum (Fig. 4E), normal rabbit serum (data not shown), or 2-keto-3-deoxyoctulonic acid (KDO), a molecule with a charge and size similar to that of 2-DN. The anti-*C. perfringens* NA antisera inhibited *C. perfringens* NA with a 50% effective dose (ED_50_) of 1:14, and the Ad-Neu3-HA-infected HEK293T cells did so with an ED_50_ of 1:67 (Fig. 4F). Even though the binding of rhNEU3 was relatively weak by surface plasmon resonance analysis (68 µM), these data clearly demonstrate that antibodies to a microbial NA, anti-*C. perfringens* NA antibodies, inhibit human NEU3 sialidase catalytic activity (see Fig. S1 in the supplemental material).

10.1128/mBio.00078-17.1 FIG S1  (A) Binding of rNeu3 to anti-*C. perfringens* NA IgG fraction. The Biacore T200 instrument was used with CM5 chips. The IgG fraction, partially purified from antiserum to *C. perfringens* NA generated in rabbits on a protein G column, was covalently coupled to the CM5 surface with the Biacore amine coupling kit in accordance with the manufacturer’s recommendations. Coupling to the surface was to 390 response units (RU). After the quenching of unbound sites on the surface with a free amine, rNeu3 was introduced over the surface at a concentration of 1 μM. The surface was then washed with Biacore HBS solution; the association and dissociation phases of the study are indicated. Pink and black curves show actual binding and a calculated fit, respectively. The binding curve is consistent with an apparent binding affinity of 1 μM. (B) Binding of rNeu3 to affinity-purified anti-rNeu3 antibody. Antibodies were covalently coupled to the CM5 chip surface as in slide 1 to a final level of 160 RU. After the quenching of unbound sites on the surface, rNeu3 was introduced at different concentrations. The pink curve shown, generated at 500 nM rNeu3, is the actual binding. The black curve shows the best fit, with an apparent *K*_*D*_ of 68 μM. The fit is reliable, as the chi-square value is <1.0. Download FIG S1, PDF file, 0.1 MB. Copyright © 2017 Feng et al. 2017 Feng et al. This content is distributed under the terms of the Creative Commons Attribution 4.0 International license.

### Anti-influenza virus goat serum recognizes rhNEU3 but not rhNEU1.

**FIG 5:**
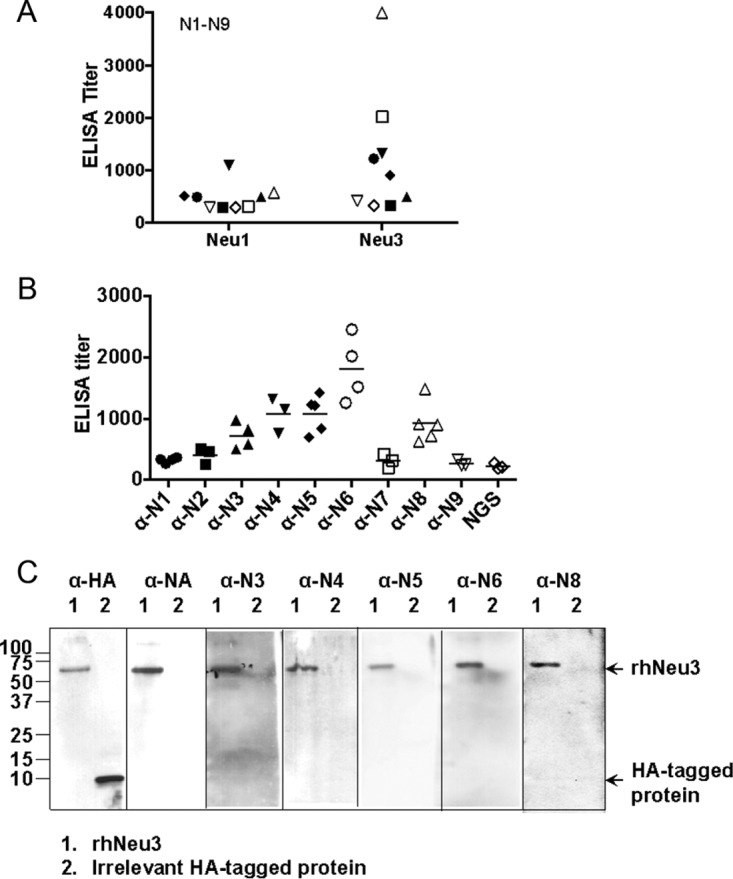
Anti-influenza virus NA antibody recognizes human NEU3. (A) Titers of antibodies to rhNEU1 or rhNEU3 in goat anti-influenza virus N1 to N9 sera were measured by ELISA. Representative data from three independent experiments are shown. (B) Titers of antibodies to rhNEU3 in specific anti-influenza virus NA serum were determined by ELISA. Data from two independent experiments were pooled. (C) rhNEU3 (lane 1) and an irrelevant HA-tagged ubiquitin protein (lane 2) were subjected to Western blotting with an anti-HA tag antibody (α-HA); rabbit anti-*C. perfringens* NA serum (α-NA); or goat anti-N3 (α-N3), -N4 (α-N4), -N5 (α-N5), -N6 (α-N6), or-N8 (α-N8) serum. Arrows indicate the expected sizes of the rhNEU3 and HA-tagged proteins.

Since NAs across species share functional and sequence homology (3, 10), we asked whether this cross-reactivity phenomenon is limited to bacterial NA and human sialidase proteins. As anti-*C. perfringens* NanI cross-recognizes human NEU3 (Fig. 1 to 3), we therefore tested for a similar antibody cross-reactivity between influenza virus NA and human sialidases. Of the 144 total combinatorial possibilities, only three HAs and two NAs in only three combinations (H1N1, H2N2, and H3N2) have ever been found in truly human-adapted influenza viruses (21). Nine serotypes of NA (N1 to N9) have been identified in avian influenza virus (22), with H5N1 being the first evidence of direct transmission of an avian influenza virus to humans. We obtained polyclonal sera against N1 to N9 from BEI Resources, Manassas, VA (see Acknowledgments) and tested their titers against rhNEU1 and rhNEU3 by ELISA. Minimal cross-reaction of these antisera with rhNEU1 was evident, but antisera to several NA serotypes displayed cross-reactivity with rhNEU3 (Fig. 5A), particularly anti-N3, -N4, -N5, -N6, and -N8 antisera (Fig. 5B). Because rhNEU3 contains an HA tag, an epitope from influenza virus HA, we examined whether the goat anti-NA antisera recognized authentic Neu3 and not the affiliated HA epitope tag. Western blot analysis of rhNEU3 and an irrelevant HA-tagged protein (ubiquitin) revealed that the anti-HA antibody identified both rhNEU3 and the HA-tagged protein (Fig. 5C). However, the rabbit anti-*C. perfringens* NA and goat anti-N3, -N4, -N5, -N6, and -N8 antisera recognized only rhNEU3 and not HA-tagged ubiquitin. Thus, like anti-*C. perfringens* NA, the goat anti-influenza virus NA antibodies cross-recognize human NEU3 but not the HA tag on either rhNEU3 or rhNEU1.

## DISCUSSION

An antibody raised to a microbial NA recognized a human endogenous mammalian sialidase, NEU3, and inhibited its enzymatic activity (Fig. 1, 3, and 4). The anti-NEU3 activity appeared to reside in a fraction of the total anti-*C. perfringens* NA response, since much of the anti-*C. perfringens* NA antibody was not bound by recombinant NEU3 on an affinity column (Fig. 1D). The *C. perfringens* NA isoform responsible for the NEU3 cross-recognition in this study appeared to be NanI (Fig. 2A and C). The anti-*C. perfringens* NA antibody bound to at least five linear epitopes in rhNEU3 (Fig. 3). Thus, the anti-*C. perfringens* NA antibody, which recognized a sialidase expressed on the surface of mouse and human leukocytes in previous studies, was presumably directed against NEU3 (4, 5). We also found that polyclonal antibodies raised to some influenza virus NAs recognized human sialidases. At least two of the antisera raised against the nine serotypes of influenza virus NA (i.e., anti-N6 and anti-N5 antisera) recognized human NEU3 but not NEU1. Interestingly, these NA types are not in current influenza vaccines. Thus, NEU3 is recognized by antibodies generated to bacterial and viral NAs.

NEU3 is considered to be a plasma membrane-bound sialidase with a substrate preference for gangliosides such as those found in abundance on the cell surface (13). NEU3 has been localized to lipid raft and caveolin-rich microdomains on the plasma membrane, where it functions as a regulator of transmembrane signaling for many cellular processes (23–25). It also has been proposed that NEU3 is involved in neuronal development, as well as in cancer metastases (26, 27). In contrast, NEU1 has been considered a lysosomal enzyme present in a complex with cathepsin A and β-galactosidase (9, 12); however, recent reports from our laboratory and those of others have also found NEU1 on the plasma membrane, but it could have been translocated to the cell surface following exocytosis (6, 28, 29). We recently observed that Neu3, but not Neu1, is expressed in human PMNs (S. Goldblum, personal observation).

Since eukaryotic NAs/sialidases are important components of the innate immune response, the cross-reactivity among microbial and eukaryotic NAs/sialidases may have clinical relevance. Endogenous sialidase activity plays a critical role in the activation of PMNs (6), monocytes (30, 31), dendritic cells (32), and endothelial cells (33), as well as T lymphocytes (34), and promotes the trafficking of leukocytes to inflammatory sites (4, 5). Moreover, we recently reported that removal of sialyl residues by sialidases from both β_2_-integrin and Toll-like receptor 4 (TLR4) enhanced their functional activity (7, 35). We recently found that both human airway epithelial cells express sialidase activity (19, 36), predominantly NEU1 but also NEU3 (37), and that signaling through epithelial cell MUC1 and the epidermal growth factor receptor is mediated in part through NEU1 (19). Since these endogenous sialidases appear to have an important physiologic role, inhibition of endogenous sialidase activity may have a deleterious effect on host immune responses, as previously speculated (38). For example, NA inhibitors used for the treatment of influenza, may also alter endogenous sialidase function, as we (Fig. 4D) and others (39) have shown. Until the role of host sialidases in the human immune response is better defined, we must be open to the possibility that the therapeutic inhibition of microbial sialidase activity may be associated with potentially deleterious consequences for the host.

These data extend the previously described microbial superfamily of NAs to include eukaryotic sialidases as well (3). The extension of the NA superfamily to eukaryotic cells is yet another example of how microbial pathogens subvert host defenses by mimicking important host defense mechanisms or physiologic functions. Thus, the host may be forced to fight off the invading pathogen but at the same time provoke collateral damage to itself. In this regard, sialic acid, which is present on virtually every cell, including immune cells, appears to play a particularly important role. For example, since developing neurons are covered with polysialic acid, two neuroinvasive bacteria, *E. coli* K1 and group B meningococci, have clothed themselves in polysialic acid capsules to escape immune recognition (40). The development of vaccines against these clinically important pathogens has been hindered by a concern that the generation of antibodies against the capsular polysaccharides of these pathogens could result in autoimmune disease.

In view of our recent reports of an important role for sialidases in innate immune cell regulation and their presence in airway epithelia, it is important that we better define the role of these enzymes in the host immune response to infections. Only then will we be able to balance the relative risks and benefits of treating mucosal pathogens, many of which express sialidases, particularly influenza virus, with therapies directed against proteins. Alternatively, by removal of sialyl residues from sialylated molecules on the cell surface, microbial NAs/sialidases may promote an excessive inflammatory response; desialylation of dendritic cells resulted in a highly significant proinflammatory cytokine response increase upon subsequent exposure to lipopolysaccharide (LPS) *in vitro*, while instillation of NA into murine lungs markedly exacerbated LPS-induced lung injury (32, 41). Since we have shown that preexposure of TLR4 to NA “primes” the host for a more robust proinflammatory response (7, 30, 35, 41), existing anti-NA interventions may be found to have a more expansive role in the treatment of pulmonary hyperinflammatory conditions. Thus, recognition of this relationship between microbial and eukaryotic NAs/sialidases presents both cautionary considerations and potential opportunities in the treatment of human inflammatory conditions.

## MATERIALS AND METHODS

### Reagents.

NA from *C. perfringens* (type V), KDO, 4-MUNANA, complete Freund’s adjuvant (CFA), incomplete Freund’s adjuvant (IFA), and anti-FLAG antibody (clone M2) were purchased from Sigma-Aldrich (St. Louis, MO). Anti-HA tag antibody was purchased from Roche Applied Science (Indianapolis, IN). Anti-NEU1 antibody was purchased from Rockland (Gilbertsville, PA). Zanamivir was purchased from GlaxoSmithKline (Research Triangle Park, NC). Oseltamivir phosphate (Tamiflu) was purchased from Sequoia Research Products (Berkshire, United Kingdom). 2-DN was purchased from Calbiochem (Gibbstown, NJ). Goat antiserum against influenza virus serotypes N1 to N9 was obtained from BEI Resources (Manassas, VA).

### Animal and immunization.

Under a University of Maryland, Baltimore, IACUC-approved protocol (number 0508004), rabbits were immunized with NA in CFA and then subjected to multiple immunizations with NA in IFA and saline to generate hyperimmune rabbit serum as previously described (4).

### Antibody purification.

Total IgG was purified from hyperimmune rabbit serum with the MAbTraP kit (GE Healthcare, Piscataway, NJ) in accordance with the manufacturer’s recommendation. Briefly, the HiTrap protein G HP column was washed with 5 ml of distilled water and equilibrated with 3 ml of binding buffer before the sample was applied. Rabbit serum was diluted with an equal volume of binding buffer and applied to the prepared column. The column was washed with 10 ml of binding buffer, and bound antibodies were eluted with elution buffer and collected. The antibodies were washed with phosphate-buffered saline (PBS) with Amicon Ultra centrifugal filter devices (Millipore, Billerica, MA). In some experiments, the purified IgG was further separated with an rhNEU3 affinity column (purchased from PrimmBiotech, Cambridge, MA, or prepared with HiTrap NHS-activated HP [GE Healthcare] in accordance with the manufacturer’s recommendation). The bound and unbound antibodies were collected separately, washed, and concentrated in PBS with the Amicon Ultra centrifugal filter devices (Millipore, Carrigtwohill, Ireland).

### Recombinant proteins and NEU3 peptides.

Human *NEU1* or *NEU3* cDNA was synthesized by PrimmBiotech (Cambridge, MA) by using sequences deposited in GenBank (accession no. NM_000433 and NM_006656), after which the 3×FLAG and HA tag sequences were inserted prior to the stop codon, respectively, and cloned into an expression vector. Recombinant HA-tagged NEU3 (rhNEU3) and FLAG-tagged NEU1 (rhNEU1) were expressed in *E. coli* as a fusion protein with an N-terminal 10×His tag and purified with a HIS affinity column (PrimmBiotech). Human NEU3 15-mer peptides with four or five amino acids overlapping were synthesized by PrimmBiotech.

### rhNEU3 affinity column preparation.

An rhNEU3 affinity column was prepared with HiTrap NHS-activated HP (GE Healthcare) in accordance with the manufacturer’s recommendation. Briefly, rhNEU3 was buffer exchanged with coupling buffer (0.2 M NaHCO_3_, 0.5 M NaCl, pH 8.3) and adjusted to 2 mg/ml. The protein solution was injected into the column and incubated for 30 min at room temperature for coupling. The column was washed, and excess active groups were deactivated with alternative injection of buffer A (0.5 M ethanolamine, 0.5 M NaCl, pH 8.3) and buffer B (0.1 M sodium acetate, 0.5 M NaCl, pH 4) three times, with a 30-min room temperature incubation after the second buffer A injection. The column was finally washed with PBS before use.

### Recombinant Ad construction.

The Ad-NEU1-FLAG, Ad-NEU3-HA, and control Ad-GFP viruses were generated with the AdEasy Adenoviral Vector System (Stratagene, La Jolla, CA) as recommended by the manufacturer. Briefly, the FLAG-tagged *NEU1* or HA-tagged *NEU3* cDNA was inserted into the pShuttle-IRES-hrGFP-1 vector by restriction enzyme digestion and ligation. The shuttle vector with *NEU1* or *NEU3* inserted or the empty shuttle vector was linearized and cotransformed with the pAdEasy-1 vector into BJ5183 competent cells to generate recombinant plasmid Ad-*NEU1*-FLAG, Ad-*NEU3*-HA, or Ad-GFP. The recombinant plasmids were extracted and confirmed by PacI digestion, followed by agarose gel analysis. The plasmids with correct recombination were further transformed into XL10-Gold cells for amplification. Sufficient amounts of plasmids were transfected into AD-293 cells with Lipofectamine (Invitrogen, Carlsbad, CA) for virus packaging. The primary virus stocks were further amplified in AD-293 cells, and their titers were quantified with a plaque assay.

### Sialidase activity assay.

Sialidase activity was assayed by using 4-MUNANA (Sigma) as the substrate as previously described (4). Lysates of transfected cells were suspended in 200 μl of 50 mM sodium acetate buffer (pH 4.4) containing 0.1% Triton X-100, protease inhibitor cocktail (Sigma), and 25 μl of 4-MUNANA (2 mM). The mixtures were briefly vortexed and incubated for 1 h at 37°C. For the 4-MUNANA assay, the reaction was terminated with 1 ml of glycine buffer (pH 10.3) containing 0.133 M glycine, 60 mM NaCl, and 42 mM Na_2_CO_3_. After the mixtures were centrifuged, the supernatants were collected and dispensed into a 96-well microtiter plate (Costar) to measure the fluorescence intensity by Fluoroskan Ascent FL (Thermo Electron Corporation). The amount of hydrolyzed 4-MUNANA in each sample was interpolated with the intensities from a serial dilution of a known concentration of 4-MUNANA (Sigma) with GraphPad Prism 4 (GraphPad Software, Inc., La Jolla, CA). In experiments using antibody immunoblockade or pharmacological inhibition, the cell lysates in the reaction buffer were incubated with heat-inactivated immune serum or inhibitor at the dilution indicated for 15 min at room temperature prior to substrate introduction.

### Western blotting.

Lysates of Ad-NEU3-HA-, Ad-NEU1-FLAG-, or Ad-GFP-infected cells, as well as rhNEU3, rhNEU1, NA, and HA-tagged ubiquitin (R&D Systems), were processed for immunoblotting. The recombinant proteins or cell lysates were resolved by SDS-PAGE (4 to 15% Tris-HCl gels; Bio-Rad Laboratories, Hercules, CA) and transferred to a polyvinylidene difluoride membrane (Millipore, Bedford, MA). The membrane was blocked with 3% bovine serum albumin in TBS and blotted with the sample serum or specific antibody, followed by a horseradish peroxidase (HRP)-conjugated secondary antibody. The membranes were washed and developed with SuperSignal West Pico Chemiluminescent Substrate (Thermo Scientific).

### ELISA.

Antibody titers were quantified by ELISA with *C. perfringens* NA, rhNEU1, or rhNEU3 as the antigen. Preimmune serum, normal rabbit IgG, or normal goat serum was used as the respective negative control. In brief, diluted serum samples in 10% milk--PBS were applied to antigen (5 μg/ml)-coated plates and incubated for 1 h at 37°C. Specific antibodies were revealed with HRP-labeled goat anti-rabbit IgG (Roche Applied Science) or rabbit anti-goat IgG (Kirkegaard & Perry Laboratories, Inc., Gaithersburg, MD), respectively, followed by TMB microwell peroxidase substrate (Kirkegaard & Perry Laboratories, Inc.). The reaction was stopped by 1 M H_2_PO_3_, and the *A*_450_ was measured. Endpoint titers were calculated through linear regression as the inverse of the serum dilution that produced an absorbance value of 0.2 above the blank (and expressed as an ELISA titer). In one particular experiment, human NEU3 peptides (5 μg/ml) were used to coat H4 plates (Costar) in carbonate buffer (pH 9.0) overnight. The *A*_450_s measured are shown.

### Structure model.

A BLAST search using the protein sequence for NEU3 (UniProt Q9UQ49) was performed against the Research Collaboratory for Structural Bioinformatics Protein Data Bank (http://www.rcsb.org) (42). A sequence alignment of human NEU3 and NEU2 was performed with Clustal W (43). A model of NEU3 was then made with the software programs SWISS-Model (44) and Phyre (45) on the basis of the crystal structures of NEU2 (PDB codes 1SO7 [sugar-induced form], 1VCU [inhibitor DANA form], and 1SNT [apo form]) (46). The PyMOL program (47) was used for model visualization and image rendering.

### Statistical analyses.

Comparison of two groups was performed by Student’s *t* test for the comparison of nonpaired samples. All results with a *P* value of <0.05 were considered statistically significant.
