# Supplementary material for: Antibody against Microbial Neuraminidases Recognizes Human Sialidase 3 (NEU3): the Neuraminidase/Sialidase Superfamily Revisited
Source: mBio. 2017 Jun 27;8(3):e00078-17. doi: 10.1128/mBio.00078-17 (PMC5487728; doi:10.1128/mBio.00078-17)
Supplement: FIG S1 [file mbo003173351sf1.pdf]

Appendix

A. Binding of rNeu3 to anti-CP NA IgG fraction

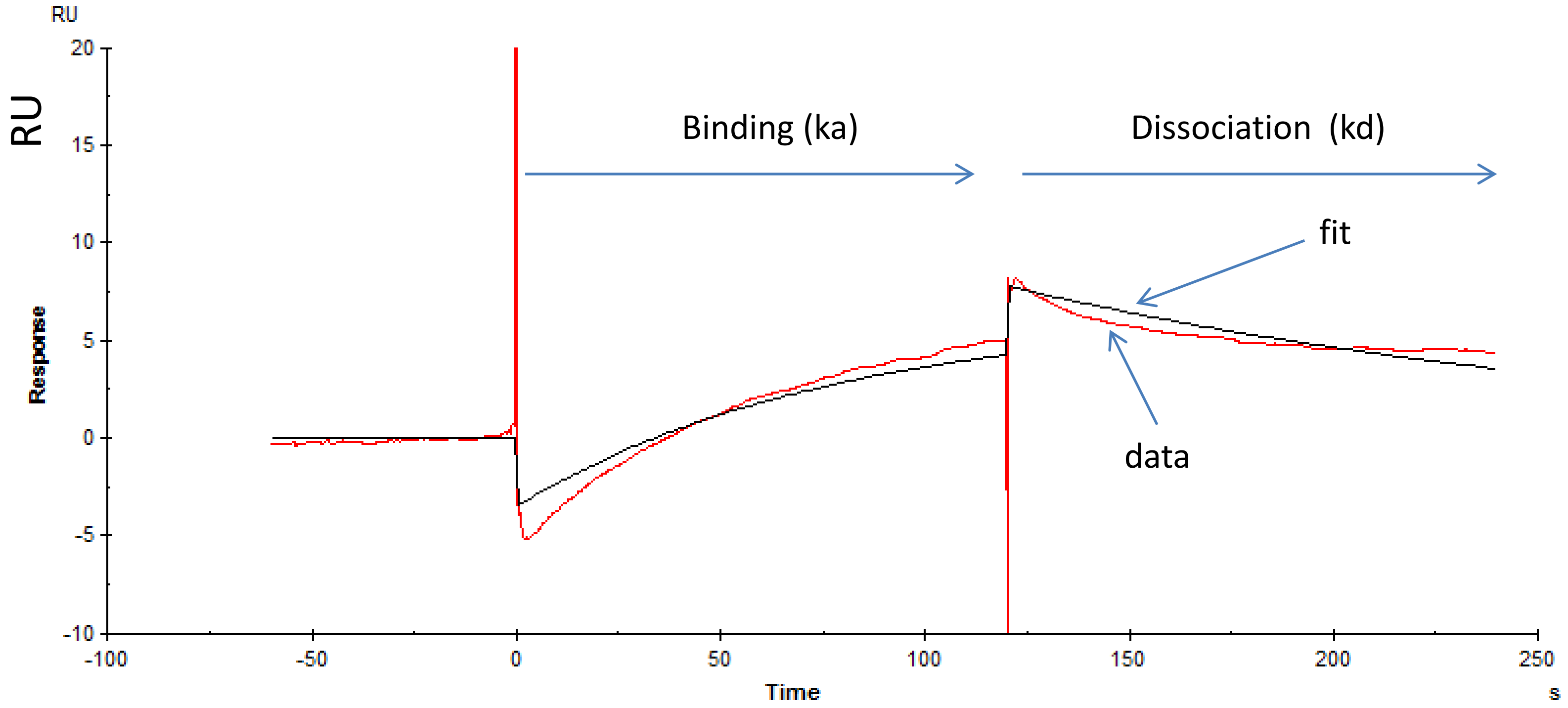

B. Binding of rNeu3 to affinity-purified rabbit antibody

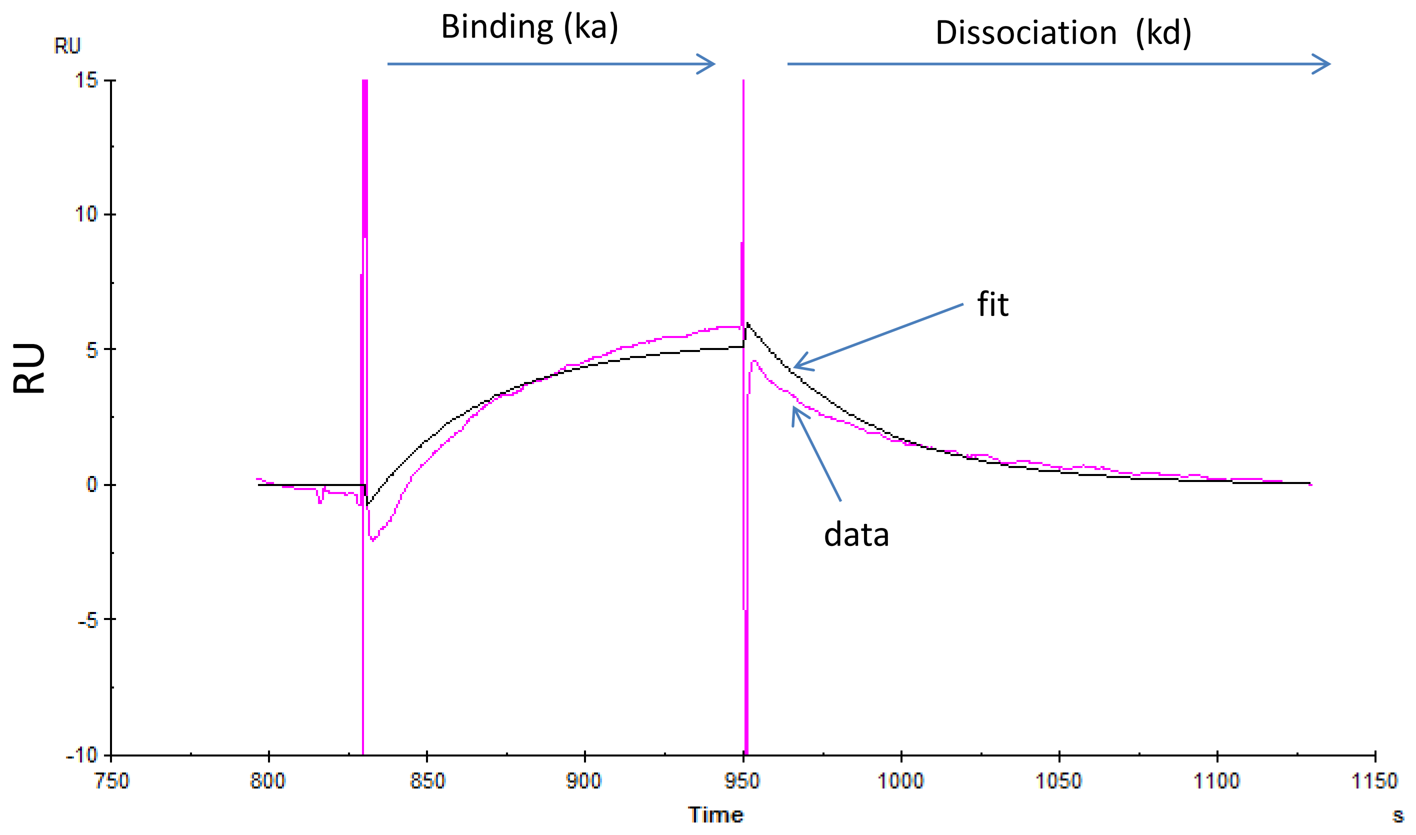

Appendix Figure Legend

A: Binding of rNeu3 to anti-CP NA IgG fraction.
The Biacore T200 instrument was used with CM5 chips. The IgG fraction, partially Purified from antiserum generated in rabbits to CPNA on a protein G column, was covalently coupled to the CM5 surface with Biacore's Amine Coupling Kit, following the manufacturer's recommendations. Coupling to the surface was to 390 RU. After quenching unbound sites on the surface with a free amine, rNeu3 was introduced over the surface at a concentration of 1 µM. The surface was then washed with Biacore's HBS solution; the association and dissociation phases of the study are indicated. Pink and black curves show actual binding and a calculated fit, respectively. The binding curve is consistent with an apparent binding affinity of 1 µM.

B: Binding of rNeu3 to affinity-purified anti-rNeu3 antibody. Antibodies were covalently coupled to the CM5 chip surface as in slide #1 to a final level of 160 RU. After quenching unbound sites on the surface, rNeu3 was introduced at different concentrations. The pink curve shown, generated at 500 nM rNeu3, is actual binding. The black curve gives the best fit, with an apparent KD = 68 µM. The fit is reliable, as the chi square value is <1.0.
